# *APC* mosaicism in a young woman with desmoid type fibromatosis and familial adenomatous polyposis

**DOI:** 10.1007/s10689-018-0072-8

**Published:** 2018-01-24

**Authors:** Astrid Tenden Stormorken, Thomas Berg, Ole-Jacob Norum, Toto Hølmebakk, Kristin Aaberg, Sonja E. Steigen, Eli Marie Grindedal

**Affiliations:** 1 Section of Inherited Cancer, Department of Medical Genetics, Oslo University Hospital, Oslo, Norway; 2 Clinical Pathology, University Hospital of North Norway, Tromsø, Norway; 3 Division of Orthopaedic Surgery, Department of Orthopaedic Oncology, Oslo University Hospital, Oslo, Norway; 4 Department of Abdominal and Paediatric Surgery, The Norwegian Radium Hospital, Oslo University Hospital, Oslo, Norway; 5 Department of Medical Biology-Tumor Biology Research Group, Faculty of Health Sciences, UiT The Arctic University of Norway, Tromsø, Norway

**Keywords:** Desmoid type fibromatosis, Familial adenomatous polyposis, *APC* gene, Mosaicism, Desmoids

## Abstract

Familial adenomatous polyposis (FAP) is usually caused by germline mutations in the adenomatous polyposis coli (*APC*) gene. The classic form is characterized by hundreds to thousands of adenomas in the colorectum and early onset colorectal cancer (CRC) if left untreated. FAP is also associated with multiple extra-colonic manifestations such as gastroduodenal polyps, osteomas, epidermoid cysts, fibromas and desmoids. Most desmoid tumours in FAP patients occur intra-abdominally. Approximately 15–20% of the *APC* mutations are de novo mutations. Somatic mosaicism has been reported in some sporadic cases of polyposis but is probably an underestimated cause of the disease. This case report presents the detection of a mosaic *APC* mutation in a 26-year-old woman who as a child had been diagnosed with desmoid type fibromatosis. FAP was suggested when she presented with extensive extra abdominal fibromatosis. Our findings indicate that *APC* mutations may be suspected in patients presenting with a desmoid regardless of its location. If there is clinical evidence that the patient has FAP, adenomas and colonic mucosa in addition to leukocyte DNA should be included in the screening, preferably using methods that are more sensitive than Sanger sequencing.

## Introduction

Familial adenomatous polyposis (FAP) is a rare autosomal dominant disorder caused by mutations in the Adenomatous Polyposis Coli (*APC)* gene. The classic form of FAP is characterized by the development of hundreds to thousands of adenomas in the colon and rectum from a young age [1]. Without surgical intervention patients have an almost 100% risk of developing colorectal cancer (CRC) before the age of 40–50 years [2]. Gastric fundic gland polyps are present in most FAP patients but rarely progress to cancer [3]. Duodenal adenomas are found in a majority of patients. Prospective studies evaluating the effect of endoscopic screening have showed a 5–18% lifetime risk for duodenal cancer [4–6]. There is a slightly increased risk of other malignancies such as cancer of the thyroid, liver, pancreas and in the central nervous system. FAP is also associated with extra-gastrointestinal manifestations such as dental abnormalities, osteomas and soft tissue tumours like epidermoid cysts, Gardner fibromas and desmoid tumours [7].

Attenuated FAP (AFAP) is characterized by a milder phenotype with fewer adenomas (< 100), a proximal location of colonic lesions and a later onset of both adenomas and CRC [8]. Extra-gastrointestinal manifestations are rare [8].

Approximately 15–20% of FAP cases are caused by de novo *APC* mutations [9] and 20% of these have somatic mosaicism [10]. The extent of both colonic and extra-colonic manifestations depends on the degree and distribution of the mutation in the different tissues. Somatic mosaicism is difficult to detect and is probably an underestimated cause of the disease [10].

A genotype–phenotype correlation is documented in FAP, but it is not consistent. Classical or severe polyposis is associated with mutations between codons 1250–1464 and desmoids with mutations throughout the whole gene, but probably more severe after codon 1444 [11–13]. AFAP is associated with mutations before codon 157, in the alternatively spliced region of exon 9 and after codon 1595 [11–13]. There is considerable phenotypic variability within and between families. Used with caution, the correlation is one of several factors considered when deciding on follow-up, timing of surgery and type of surgery.

Ten to 30% of FAP patients have desmoids and the majority occur within the abdomen or in the abdominal wall [14–16]. Desmoids can develop at any age but the incidence is highest in the second and third decades of life [16, 17]. Several studies confirm that a family history of desmoids, previous abdominal surgery and, more debated, an *APC* pathogenic mutation at the 3′ end of the gene, are predictors for desmoid tumour development [14, 16, 17]. Most cases of desmoid type fibromatosis are sporadic; but in 5–16% they are associated with FAP [14, 15, 18].

Here we report the detection of a somatic mosaic *APC* mutation in a patient who was diagnosed with desmoid type fibromatosis in childhood. FAP was eventually suggested after she developed extensive extra-abdominal fibromatosis.

## Case presentation

A 26-year-old female was referred to the Sarcoma group at Oslo University Hospital with a large soft tissue lesion in her left thigh, described histologically as inactive fibromatosis. MRI-findings were consistent with this diagnosis. Subcutaneous soft tissue tumours were detected in her right buttock and along the spine. Histologically a surgical biopsy from one of these lesions was described as fibrous tissue but not fibromatosis. It was suggested that it could be a Gardner fibroma. The patient reported some pain in her hip and back.

She had been diagnosed with desmoid type fibromatosis as a child following removal of several subcutaneous soft tissue tumours and epidermoid cysts. The initial tumour was detected when she was 2 months old. Three tumours were removed during her first year and nine tumours were subsequently removed before she turned three. Neurofibromatosis was considered as a differential diagnosis but as she did not have any café-au-lait spots, this diagnosis was excluded. Over the years she had several small lesions removed. Biopsies revealed epidermoid cysts and fibromatous tissue.

Based on her medical history, FAP was suggested and upper and lower endoscopic examinations were performed. Some fifty adenomas were detected throughout her colon but predominantly in the distal part. Biopsies showed low-grade dysplasia. In her stomach, 50–70 fundic gland polyps and some adenomas were detected and one adenoma with low-grade dysplasia was detected in the duodenum. Phenotypically she had a mild colon polyposis. There was no history of fibromatosis, polyps or CRC in her close family. She was referred to genetic counselling and testing.

**Fig. 1:**
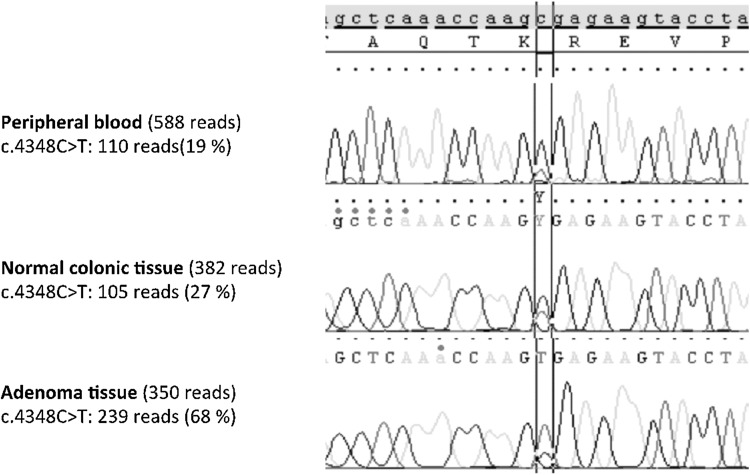
Sequence analysis of the *APC* mutation c.4348C > T (p.Arg1450*) in peripheral blood, normal colonic tissue and adenoma tissue from the patient. The fraction of NGS reads representing the c.4348C > T mutation is shown to the left

Germline testing of the *APC* gene was initially performed utilizing Sanger sequencing and Multiplex Ligation-dependent Probe Amplification Analysis. Sanger sequencing of leukocyte DNA from peripheral blood indicated presence of a pathogenic mutation, c.4348C > T (p.Arg1450*), in the *APC* gene. The signal representing the mutation was very weak, indicating somatic mosaicism. DNA was extracted from normal colonic mucosa and from adenoma tissue. Sanger sequencing revealed significantly higher levels of the mutation in these tissues compared to the blood sample (Fig. 1). After the initial investigation we were able to perform next generation sequencing (NGS) of a cancer gene panel library (Illumina TruSight cancer Sequencing panel). This showed that the frequency of the mutation varied among different tissue types, and hence confirmed somatic mosaicism of the APC mutation in the patient.

Her parents and siblings were tested but did not have the mutation, consistent with a de novo origin of the mutation.

The mutation in this patient affected codon 1450, a site of the *APC* gene associated with classical polyposis and desmoid development. Based on her high risk of CRC, prophylactic proctocolectomy was recommended, and she underwent laparoscopic proctocolectomy with an ileal pouch and ileoanal anastomosis without diverting ileostomy. She has been followed with regular endoscopic examinations of the ileal pouch and oesophagogastroduodenoscopy is performed every 6 months due to duodenal adenomas.

Abdominal MRI 9 months post surgery was unremarkable. A year later, less than 2 years after surgery, she presented with three desmoids. Two were located in the abdominal wall (laparoscopy port site and Pfannenstiel incision) and the third intra-abdominally in the mesentery.

MRI 6 and 9 months later showed desmoid progression. However, due to the risk of triggering a more aggressive course of the disease and the unlikeliness of a radical excision, a “wait and see” approach was adopted. MRI, taken almost 3 years after surgery, showed that the desmoids had remained stable. However, since then, the latest MRI has shown that the lesions have grown.

## Discussion

In FAP, soft tissue tumours like desmoids and epidermoid cysts may present before the appearance of colon polyps [15, 19]. Several studies have demonstrated that asymptomatic patients who have extra-truncal desmoids, are less likely to have FAP [14, 15, 18], and that in FAP patients, most of the desmoids are located in the abdominal cavity/abdominal wall [14–16]. Testing of the *APC* gene and/or colonoscopy to rule out FAP is therefore not performed routinely in patients with extra-abdominal desmoids. This patient initially only had extra-truncal fibromatosis and FAP was not considered before she had more extensive fibromatosis in her buttock and along her spine.

There are several reports of patients and families with desmoids, with none or a few polyps, but who harbour a mutation after codon 1900 in the 3′ end of the *APC* gene [20–22]. Our patient had her first colonoscopy at 26 years of age and had fewer colon polyps than expected according to the site of her mutation. A colonoscopy done 10–15 years earlier would perhaps have missed the diagnosis, as she may not have developed polyps at that time. Our case illustrates that FAP may be suspected in patients with only extra-abdominal desmoids, and that not only colonoscopy but also genetic testing of the *APC* gene should be offered to these patients. Previous studies have already suggested testing for *APC* mutations in children with extracolonic manifestations as hepatoblastoma, osteoma and Gardner fibroma [11, 19, 23].

A de novo *APC* gene mutation was anticipated, as there was no family history of desmoids, polyps or CRC. Sanger sequencing revealed very weak signals representing the mutation in leukocyte DNA, and could have been missed. However, since mosaicism is not infrequent in FAP, sequencing DNA from one of her adenomas and her colonic mucosa followed up the analyses. This revealed stronger signals representing the mutation, suggests an early postzygotic mutational event involving both the endoderm (adenoma) and the mesoderm (blood) and shows the importance of including adenomas and colonic mucosa in the screening. Other screening methods including NGS have shown higher sensitivity than Sanger sequencing in detecting low level *APC*-mutation mosaicism [10, 24, 25]. A substantial number of mosaic cases may be missed when doing Sanger sequencing of blood (leukocyte DNA) [10, 24, 25]. Several studies have demonstrated that adenomas should be included in the screening for low level *APC* mosaicism as a high frequency of cases are not detectable in leukocyte DNA [10, 24, 25]. A recent publication underlines the importance of testing multiple adenomas as co-occurence of sporadic adenomas within a mosaic environment has been reported [26]. Our study also highlights the power of NGS in identifying and characterizing somatic mosaicism in various tissues.

The *APC* gene mutation in our patient affecting codon 1450 is in the mutation cluster region of the *APC* gene. This position is at the site for classical FAP, desmoids, osteomas and epidermoid cysts. Despite this, the patient had an attenuated colon polyposis with approximately 50 colon adenomas. But contrary to AFAP, the adenomas had a predominantly distal location in the colon. Her upper gastrointestinal findings were in accordance with what can be seen both in FAP and AFAP. Several studies have shown that somatic mosaicism for *APC* pathogenic variants usually associated with classic FAP may express a milder colon polyposis phenotype [10, 24, 25]. In these studies, there was only one patient with a desmoid tumour [10]. In our patient, the first fibromatous lesion was noted when she was 2 months old; she had several others over the years and massive fibromatosis in her thigh, buttock and back at the time she was diagnosed with FAP. Some reports have shown a higher incidence of epidermoid cysts and a more severe desmoid disease in patients with a mutation between codon 1445 and codon 1580 than in patients with mutations in other regions of the *APC* gene [27–29]. Her mild colon polyposis and severe fibromatosis may therefore be explained by varying distribution of the mutation in different tissues. Additional genetic and environmental factors may also have an influence.

Prophylactic surgery at an early age is recommended in patients with FAP. Surgery is known to be a trigger of desmoid formation, and postponing surgery in patients at risk of developing desmoids could be advocated. In our opinion, such a policy cannot be justified as malignant transformation can occur even under close surveillance. Unfortunately, our patient developed intra-abdominal and abdominal wall desmoids shortly after surgery and she is now in a “wait and see” situation as desmoids can regress, remain stable or progress, in which case medical treatment could be considered.

Although identification of an *APC* mutation confirmed a FAP diagnosis for this patient, genetic counselling in this situation is difficult. For patients carrying germline mutations, offspring will have a 50% chance of inheriting the mutation. However, when there is somatic mosaicism the risk depends on the level of mosaicism in the patient´s germ cells [10, 30]. Germline mosaicism in an unaffected parent of children with colonic adenomatous polyposis has been reported [10, 31]. Another counselling issue is that the offspring will most likely have a more severe phenotype than the affected parent [25].

We counselled our patient not to become pregnant and give birth, primarily because her pelvic pouch would probably necessitate a caesarean section, which would put her at increased risk of abdominal desmoids. The natural history of her disease has proved this to be sound advice.

## Conclusion

Our case illustrates that a history of extra-abdominal fibromatosis may be an indication for colonoscopy and *APC* genetic testing, and that *APC* mosaicism may be the cause of not just single cases of polyposis but also of the extra-colonic features of FAP. Adenomas and colonic mucosa in addition to leukocyte DNA should be included in the screening, preferably using NGS, if there is a suspicion of FAP.
